# Assessment of Maternal Genetic Diversity and Mitochondrial Population Structure of Endangered Indigenous Chicken Breeds in China

**DOI:** 10.3390/ani16121933

**Published:** 2026-06-22

**Authors:** Wanqiang Chen, Xiujun Tang, Yanfeng Fan, Jing Zhang, Mengjun Tang, Lina Ma, Yushi Gao, Xiaoxu Jia

**Affiliations:** Jiangsu Institute of Poultry Science, Yangzhou 225125, China; chenwq8231@163.com (W.C.); tangxj0918@126.com (X.T.); fanyanfeng226@126.com (Y.F.); zhangjing9143@126.com (J.Z.); tangmengjun1980@163.com (M.T.); marina1986tiger@163.com (L.M.)

**Keywords:** endangered indigenous chicken, mitochondrial DNA, conservation status, genetic diversity, genetic structure

## Abstract

This study aimed to evaluate the maternal genetic diversity and mitochondrial population structure of once endangered indigenous chicken breeds in China under current conservation conditions. The genetic characteristics of six endangered indigenous chicken breeds, namely the Bian chicken, Jinyang Silky chicken, Pudong chicken, Xiaoshan chicken, Zhongshan Shalan chicken, and Pengxian Yellow chicken, were analyzed based on mitochondrial DNA (mtDNA) D-loop region sequences. Blood samples were collected from the wing vein. The D-loop region was amplified by PCR, and genetic characteristics were analyzed using bioinformatics approaches. A total of 368 individuals were amplified and sequenced, yielding complete D-loop sequences of 1231 and 1232 bp. Sequence alignment identified 42 polymorphic sites, with a hypervariable region primarily located between 167 and 446 bp. The overall haplotype diversity, nucleotide diversity, and the average number of nucleotide differences were 0.876 ± 0.010, 0.00603 ± 0.00012, and 7.426, respectively, with significant inter-breed variation. Haplotype analysis identified 32 haplotypes belonging to haplogroups A, B, C, E, F, and G. The proportion of breed-specific and shared haplotypes varied among breeds, and several high-frequency haplotypes were widely distributed across populations. Analysis of molecular variance (AMOVA) indicated that the majority of genetic variation occurred within breeds (83.41%), whereas among-breed variation accounted for 16.59% (*Fst* = 0.166), suggesting moderate population differentiation. The median-joining haplotype network exhibited a radial pattern centered on several core haplotypes, with no evidence of breed-specific clustering of maternal lineages. Neutrality tests (*Tajima’s D* and *Fu’s Fs*) yielded non-significant results, consistent with neutral evolution. However, mismatch distribution analyses suggested possible population expansion in the Zhongshan Shalan chicken and Pengxian Yellow chicken. In summary, the six endangered indigenous chicken breeds retain a moderate level of maternal genetic diversity under current conservation conditions. However, differences among breeds were observed in the maintenance of genetic diversity and population structure.

## Simple Summary

Chinese indigenous chicken breeds, which were once endangered, are now the focus of conservation efforts. This study evaluated the current maternal genetic diversity and population structure of six breeds (the Bian chicken, Jinyang Silky chicken, Pudong chicken, Xiaoshan chicken, Zhongshan Shalan chicken, and Pengxian Yellow chicken) using mitochondrial DNA D-loop sequences from 368 individuals. The results revealed that these breeds maintain a moderate level of maternal genetic diversity, with most genetic variation occurring within breeds instead of between breeds. Several haplogroups (A, B, C, E, F, and G) were identified, and the distribution of haplotypes varied across breeds. Overall, under current conservation conditions, these once endangered breeds retain appreciable genetic diversity.

## 1. Introduction

Livestock genetic resources constitute a fundamental component of agricultural biodiversity, encompassing the heritable traits of domesticated animal species shaped by long-term natural adaptation and human selection. Among these, poultry, especially domestic chickens, constitutes the most numerous and widely distributed livestock species worldwide, playing a critical role in smallholder livelihoods, rural economies, and food security in both developed and developing regions. However, despite their socio-economic importance, many indigenous chicken breeds are increasingly threatened by genetic erosion and population decline, and are therefore recognized as endangered genetic resources, requiring urgent conservation attention.

Globally, approximately 30% of poultry breeds were at risk of extinction, and 9% have already become extinct, with chickens accounting for the largest proportion of threatened breeds [1]. Rapid structural changes in the poultry industry have been identified as a major driver of this genetic erosion. In response, the Ministry of Agriculture and Rural Affairs issued the National Plan for the Conservation and Utilization of Livestock and Poultry Genetic Resources (2016–2020), which formally assessed the conservation status of several indigenous chicken breeds and incorporated them into key protection programs. According to this plan, the Yantai Cankang chicken and Northern Shaanxi chicken were declared extinct; the Pengxian Yellow chicken was classified as critically endangered; and the Jinyang Silky chicken, Bian chicken, Pudong chicken, Xiaoshan chicken, and Zhongshan Shalan chicken were categorized as endangered. The different endangered states reflected multiple pressures at that time, including population decline, erosion of genetic diversity, and changes in production systems. Nevertheless, endangerment status is dynamic and may change following the implementation of protection programs, adjustments in production systems, and improvements in genetic resource management. Therefore, continuous genetic monitoring is essential to objectively evaluate conservation outcomes, identify potential genetic risks, and optimize future conservation strategies.

Genetic diversity is a fundamental indicator of a species’ or breed’s long-term viability and evolutionary potential. High levels of genetic diversity enhance the ability of populations to adapt to environmental changes, resist disease, and maintain stable productive performance, whereas reduced diversity is often associated with increased inbreeding and diminished adaptive capacity, thereby exacerbating the risk of endangerment [2]. Therefore, the systematic assessment of genetic diversity has become a critical scientific basis for the conservation and sustainable utilization of indigenous chicken breeds. With advances in molecular biology, DNA-based markers have been widely applied in the study of poultry genetic resources [3]. Among these, mitochondrial DNA (mtDNA) is particularly suitable for population genetic analyses due to its maternal inheritance, high copy number, rapid evolutionary rate, and absence of recombination [4]. Within the mitochondrial genome, the control region (D-loop) exhibits a high mutation rate and sensitivity to short-term demographic changes, making it a widely used marker in studies of genetic diversity and phylogeography of chickens and other poultry species [5,6,7,8]. Moreover, several Chinese indigenous breeds have been demonstrated to play an important role in the global maternal origin of domestic chickens [9,10]. Despite these advances, systematic evaluations of changes in genetic diversity among endangered indigenous chicken breeds remain limited. In conservation practice, several breeds have been managed through the establishment of conservation farms and the implementation of combined in situ and ex situ strategies, resulting in partial recovery of population size. However, ex situ populations may maintain lower genetic diversity than in situ populations [11]. Furthermore, the introduction of exotic germplasm or excessive reliance on a limited number of core breeding individuals may alter genetic structure and potentially accumulate hidden genetic risks [12]. Therefore, reliance solely on census population size or production scale is insufficient to comprehensively evaluate conservation status. Integrating molecular genetic data is essential for dynamic and objective assessment of endangerment risk.

Against this background, the present study focuses on six indigenous chicken breeds classified as endangered in the 2016 Chinese national announcement. Using mitochondrial DNA markers, we systematically assessed maternal lineage diversity and mitochondrial population structure rather than genome-wide conservation status. By integrating previous research findings, we further examined the current status and potential trends in genetic diversity under existing conservation and utilization frameworks. The results aim to provide a scientific basis and empirical evidence for evaluating conservation effectiveness, optimizing protection strategies, and promoting the sustainable utilization of indigenous chicken genetic resources in China.

## 2. Materials and Methods

### 2.1. Population Survey and Sample Collection

Six endangered indigenous chicken breeds listed in the 2016 announcement of the Ministry of Agriculture and Rural Affairs, namely the Bian chicken, Jinyang Silky chicken, Pudong chicken, Xiaoshan chicken, Zhongshan Shalan chicken, and Pengxian Yellow chicken, were included in this study. Population status was investigated through a literature review, field visits, on-site measurements, genetic sampling, and interviews with managers of conservation farms.

**Figure 1:**
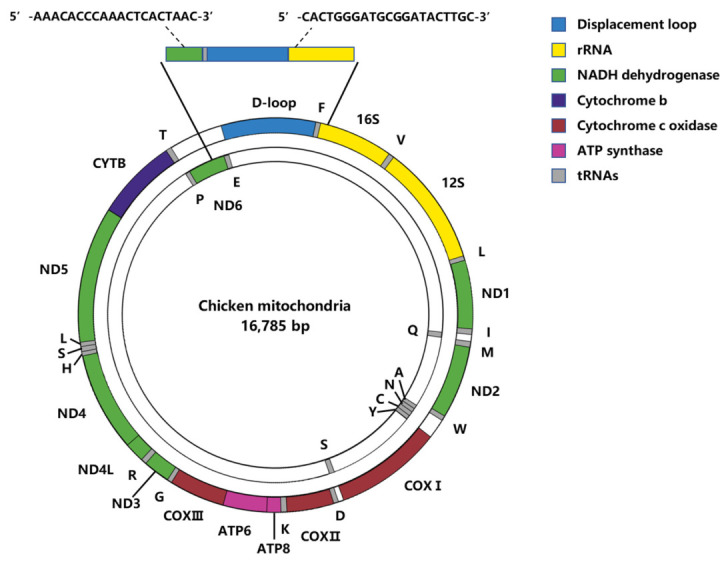
Circular structure and linear organization of chicken mitochondrial DNA.

All samples were collected from officially designated conservation units (Table 1 and Figure 1). Individuals were selected from different breeding families, breeding groups, or subpopulations within each conservation farm. Available pedigree and breeding management records provided by conservation units were consulted during sampling to reduce the probability of repeatedly sampling closely related maternal lineages. Individuals with known direct pedigree relationships or originating from the same maternal family were avoided whenever possible. Approximately 1–2 mL of blood was collected from the wing vein and stored in anticoagulant tubes at −20 °C until analysis. Genomic DNA was extracted using a commercial kit (DP308, Tiangen Biotech Co., Ltd., Beijing, China) according to the manufacturer’s instructions. DNA integrity was assessed by 1.0% agarose gel electrophoresis, and qualified DNA samples were subsequently stored at −20 °C for PCR amplification.

### 2.2. PCR Amplification and Sequencing of the Mitochondrial D-Loop Region

Primers for amplification of the mitochondrial DNA D-loop region were designed according to Jia et al. (2016) [13] (Figure 1). Forward: 5′-AAACACCCAAACTCACTAAC-3′; Reverse: 5′-CACTGGGATGCGGATACTTGC-3′. PCR reactions were performed in a total volume of 50 μL, containing 2 μL of template DNA (50–100 ng), 25 μL of 2× PCR Master Mix, 1 μL of each primer (10 μmol/L), and 21 μL of sterile distilled water. The thermal cycling conditions were as follows: initial denaturation at 95 °C for 5 min; 35 cycles of denaturation at 95 °C for 30 s, annealing at 56 °C for 30 s, and extension at 72 °C for 30 s; followed by a final extension at 72 °C for 7 min. PCR products were verified by electrophoresis on 1.2% agarose gels to confirm the expected fragment size. Primer synthesis and bidirectional sequencing of PCR products were performed by Sangon Biotech Co., Ltd. (Shanghai, China).

### 2.3. Sequence Assembly and Genetic Diversity Analysis

Raw sequencing chromatograms were manually inspected and assembled using SeqMan v11.1.0 (DNASTAR, Madison, WI, USA). Primer sequences and low-quality regions were trimmed. Only sequences with clear electropherograms and unambiguous base calls were retained for further analyses to ensure data reliability. All valid sequences were aligned using ClustalX 2.1 [14], and a consensus fragment corresponding to the complete D-loop region was obtained. Based on the aligned sequences, genetic diversity parameters were calculated using DnaSP v6.12.03 [15], including the number of polymorphic sites (*S*), the number of haplotypes (*H*), haplotype diversity (*Hd*), nucleotide diversity (*Pi*), and the average number of nucleotide differences (*K*).

### 2.4. Haplotype Network and Population Genetic Structure Analysis

Haplotypes were classified according to standard mitochondrial haplogroup definitions using the MitoToolPy script v1.0 [16] based on variable sites within the D-loop region. A median-joining (MJ) haplotype network was constructed using Popart v1.7 [17] to visualize maternal relationships and infer historical diffusion patterns among breeds. Population differentiation was assessed using Arlequin v3.5 [18]. Pairwise *Fst* values and gene flow (*Nm*) were calculated, and statistical significance was evaluated using 1000 permutations. *Tajima’s D* and *Fu’s FS* neutrality tests were conducted to assess deviations from neutrality, and mismatch distribution analyses were performed to infer historical demographic dynamics. Analysis of molecular variance (AMOVA) was applied to partition genetic variation into within-breed and among-breed components, providing an overall evaluation of population genetic structure.

## 3. Results

### 3.1. Conservation Status of Endangered Indigenous Chicken Breeds

Based on the systematic investigation of the six endangered local chicken breeds presented, each breed has experienced significant population fluctuations across different historical stages, with varying degrees of population contraction observed. During the mid-to-late 20th century, local chicken populations maintained relatively stable or even large breeding scales. However, a pronounced decline occurred in the late 20th century, with several breeds approached near-extinction status. For instance, the population of the Bian chicken plummeted from 400,000 to 500,000 individuals in the early 1970s to fewer than 5000 around 2000. The Pengxian Yellow chicken decreased sharply from about 285,000 in 1981 to merely 61 by the end of 2005. The Xiaoshan chicken, which numbered over 2 million around 1986, fell to about 10,000 in 1995 and further declined to about 1500 by 2005. These data indicate that all breeds underwent severe population contraction under the pressures of industrial restructuring in the poultry industry and the widespread introduction of commercial breeds. Regarding spatial distribution patterns, the remaining populations are mainly confined to their original breeding regions or primary production areas. Except for the Zhongshan Shalan chicken, which has formed large-scale commercial populations in multiple locations within the Pearl River Delta, most other breeds rely on single or a few conservation farms to maintain population continuity. This results in a high degree of spatial isolation among populations and limited genetic exchange between regions.

According to the Third Chinese National Survey of Livestock and Poultry Genetic Resources, all six breeds have experienced substantial recovery in census population size compared with their historical bottlenecks. With the exception of the Zhongshan Shalan chicken, the other five breeds have been included in the National List of Protected Livestock and Poultry Genetic Resources and have established national or provincial conservation farms. Since the initiation of pure-line conservation for the Zhongshan Shalan chicken in 2004, both the pure-line and commercial populations have expanded steadily. Currently, the commercial population has reached the million-level, demonstrating remarkable success in population recovery. Overall, although the current population sizes of these breeds have recovered significantly compared with their historical lows, considerable disparities persist among breeds in terms of founder population size, conservation systems development, and utilization level. Demographic recovery does not necessarily indicate the restoration of genetic diversity or long-term genetic stability. Comprehensive conservation evaluation still requires the assessment of effective population size, inbreeding levels, and genome-wide diversity through long-term genetic monitoring.

### 3.2. Sequence Characteristics and Variation in the Mitochondrial D-Loop Region

The mitochondrial DNA D-loop region was successfully amplified and sequenced in 368 individuals from the six breeds. All samples yielded clear amplification products. After the sequence assembly and quality control, 368 complete D-loop sequences were obtained, with lengths ranging from 1231 to 1232 bp. (All sequences described in the study have been deposited in GenBank. Accession numbers provided in the study range from PZ189266 to PZ189633). Among these, 265 sequences were 1231 bp and 103 were 1232 bp in length. The observed length variation was attributable to the deletion of a single cytosine within the 852–859 bp region, which consists of a homopolymeric C stretch. The alignment of the D-loop sequences identified 42 polymorphic sites across the six breeds, all of which were biallelic. Of these, 16 were singleton variable sites located at positions 199, 210, 234, 236, 239, 240, 252, 254, 317, 330, 354, 355, 396, 586, 636, and 904 bp, while 26 were parsimony-informative sites located at positions 133, 167, 212, 217, 225, 242, 243, 246, 256, 261, 281, 296, 302, 308, 310, 315, 322, 342, 363, 367, 370, 446, 686, 792, 1214, and 1215 bp. Polymorphic sites were distributed throughout the D-loop region (133–1215 bp), with a relatively high concentration between 167 and 446 bp, representing a hypervariable segment. Regarding substitution types, 40 transitions were detected, including 31 T–C and 9 A–G substitutions, as well as two transversions (A–C) located at positions 296 and 317 bp. Base composition analysis showed an average G + C content of 39.8% and an A + T content of 60.2%, indicating a pronounced A + T bias in the D-loop region.

### 3.3. Maternal Genetic Diversity

The maternal genetic diversity of the six breeds was evaluated based on D-loop sequences (Table 2). Across all populations, overall haplotype diversity (*Hd*), nucleotide diversity (*Pi*), and the average number of nucleotide differences (*K*) were 0.876 ± 0.010, 0.00603 ± 0.00012, and 7.426, respectively.

The number of polymorphic sites (*S*) ranged from 15 to 31 among breeds. The Jinyang Silky chicken exhibited the highest number of variable sites (31), whereas the Xiaoshan chicken showed the lowest (15), indicating considerable heterogeneity in mitochondrial sequence variation among breeds. Haplotype diversity (*Hd*) ranged from 0.589 to 0.840. The Jinyang Silky chicken displayed the highest haplotype diversity (*Hd* = 0.840), whereas the Pengxian Yellow chicken exhibited the lowest (*Hd* = 0.589). The remaining breeds showed intermediate levels of haplotype diversity, suggesting differential retention of maternal genetic variation under endangered conditions.

Nucleotide diversity (*Pi*) also varied among breeds, ranging from 0.0041 to 0.0069. The Jinyang Silky chicken and Zhongshan Shalan chicken showed relatively high nucleotide diversity, whereas the Bian chicken and Pudong chicken exhibited comparatively lower values. The average number of nucleotide differences (*K*) followed a similar trend to nucleotide diversity, ranging from 5.095 to 8.530 across breeds. These results indicate that although all six breeds retain measurable maternal genetic variation, substantial differences exist in the extent of genetic diversity maintained among them.

### 3.4. Haplotype Composition and Distribution

The haplotype composition of the six breeds showed marked heterogeneity in the haplotype number, proportion of breed-specific haplotypes, and frequency of shared haplotypes (Table 3). Zhongshan Shalan chicken exhibited the highest number of haplotypes (14), followed by Jinyang Silky chicken (12), whereas the Bian chicken and Xiaoshan chicken each possessed only six haplotypes. These differences indicate variation in the accumulation of maternal genetic variation among breeds.

In terms of breed-specific haplotypes, Jinyang Silky chicken displayed the highest proportion, with seven unique haplotypes accounting for 60% of individuals. The Xiaoshan chicken also showed a relatively high proportion of unique haplotypes (40%, three haplotypes). In contrast, the Bian chicken, Pudong chicken, and Pengxian Yellow chicken exhibited low proportions of unique haplotypes. Only one unique haplotype (3.3%) was detected in Bian chicken, and most individuals were assigned to shared haplotypes. The proportion of individuals carrying shared haplotypes exceeded 85% in Bian chicken, Pudong chicken, and Pengxian Yellow chicken, reaching 96.7% in Bian chicken. Although Zhongshan Shalan chicken harbored a relatively large number of haplotypes, 88.9% of individuals belonged to shared haplotypes.

Haplogroup classification showed that the 32 haplotypes identified across the six breeds belonged to haplogroups A, B, C, E, F, and G. However, the proportional composition of haplogroups differed among breeds. The Bian chicken was predominantly assigned to haplogroup E (70%), whereas Pudong chicken and Pengxian Yellow chicken were mainly distributed in haplogroup A (67% and 61%, respectively). Xiaoshan chicken showed a more dispersed pattern across haplogroups A, C, and E. In contrast, Jinyang Silky chicken and Zhongshan Shalan chicken exhibited more complex haplogroup structures: the Jinyang Silky chicken had relatively high representation in haplogroups A, B, and G, whereas the Zhongshan Shalan chicken was primarily composed of haplogroups B and E.

**Figure 2:**
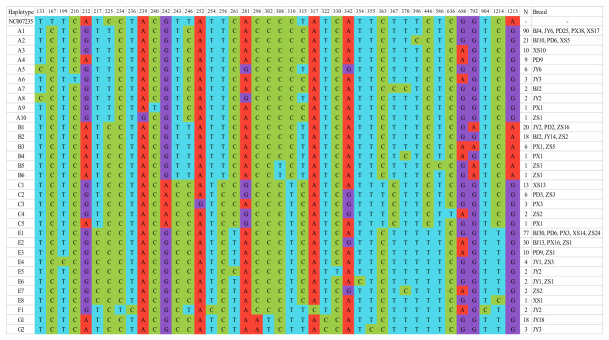
Mutation sites and breed distribution of haplotypes in six Chinese endangered indigenous chicken breeds.

Among the 32 haplotypes detected (Figure 2), haplotype A1 was the most frequent (90 individuals), followed by E1 (77 individuals). These high-frequency haplotypes were distributed across multiple breeds, with some occurred at relatively high frequencies in geographically distinct populations, thereby constituting dominant maternal lineages within the overall sample. Haplogroups F and G were detected exclusively in Jinyang Silky chicken, with haplotype G1 represented by 18 individuals.

### 3.5. Population Genetic Structure and Differentiation

The results of analysis of molecular variance (AMOVA), pairwise *Fst*, and gene flow (*Nm*) are presented in Table 4 and Table 5. AMOVA indicated that 83.41% of the total genetic variation occurred within breeds, whereas 16.59% was explained by variation among breeds. The overall fixation index was *Fst* = 0.166, suggesting moderate maternal genetic differentiation among the six endangered breeds. By calculating *Fst* values, the degree of genetic differentiation among different breeds was quantified, providing a basis for inferring population genetic structure and historical gene exchange events.

Pairwise *Fst* values revealed substantial heterogeneity in genetic differentiation among breed pairs. The lowest *Fst* value was observed between Pudong chicken and Pengxian Yellow chicken (*Fst* = 0.005), followed by Xiaoshan chicken versus Pengxian Yellow chicken (0.035) and Xiaoshan chicken versus Pudong chicken (0.041). In contrast, the highest *Fst* value occurred between Bian chicken and Jinyang Silky chicken (0.245), followed by Bian chicken versus Pudong chicken (0.238).

Patterns of gene flow (*Nm*) were consistent with *Fst* estimates. Notably, Pudong chicken and Pengxian Yellow chicken exhibited extremely high gene flow (*Nm* = 92.659), indicating low maternal genetic differentiation between these two breeds. Overall, these results demonstrate varying degrees of maternal genetic connectivity and differentiation among endangered indigenous chicken breeds.

### 3.6. Haplotype Network and Phylogenetic Relationships

**Figure 3:**
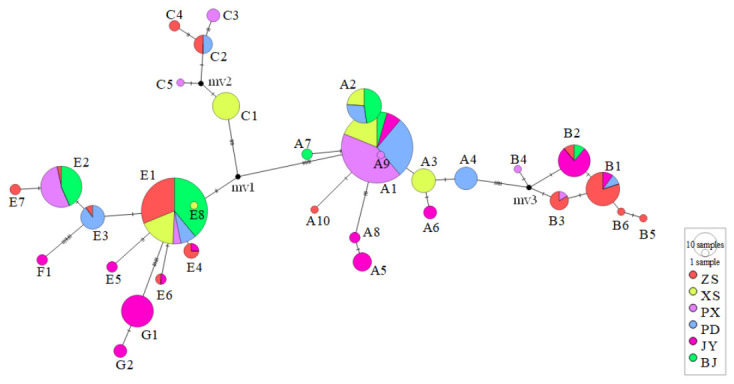
Median network of mtDNA D-loop haplotypes in six Chinese endangered indigenous chicken breeds ^(1)^. ^(1)^ The circle sizes are proportional to haplotype frequencies. Different shades of the circles correspond to distinct populations.

The median-joining haplotype network is shown in Figure 3. Haplogroups A, B, C, E, F, and G comprised 145, 47, 25, 128, 2, and 21 sequences, respectively. The network displayed a star-like topology centered on several core haplotypes, from which peripheral haplotypes radiated. Several large nodes representing high-frequency shared haplotypes were widely distributed among breeds, suggesting that these haplotypes correspond to ancestral or historically widespread maternal lineages. The branches connecting haplogroups were separated by multiple mutational steps; however, overall branch lengths were relatively short, and intermediate haplotypes were limited. Individuals from different breeds were distributed across multiple haplogroups without forming clear breed-specific clusters, indicating weak maternal lineage differentiation among breeds. This pattern is consistent with the moderate population differentiation inferred from *Fst* analysis.

Within individual breeds, both core shared haplotypes and low-frequency peripheral haplotypes were observed. The presence of peripheral haplotypes suggests ongoing accumulation of breed-specific maternal variation, whereas concentration around central haplotypes in certain breeds may reflect historical changes in effective population size or restricted maternal sources during conservation and breeding processes. Importantly, the presence of shared haplotypes in the network does not necessarily indicate recent introgression but instead reflects a common maternal genetic foundation established during domestication or long-term artificial selection.

### 3.7. Demographic History Analysis

**Figure 4:**
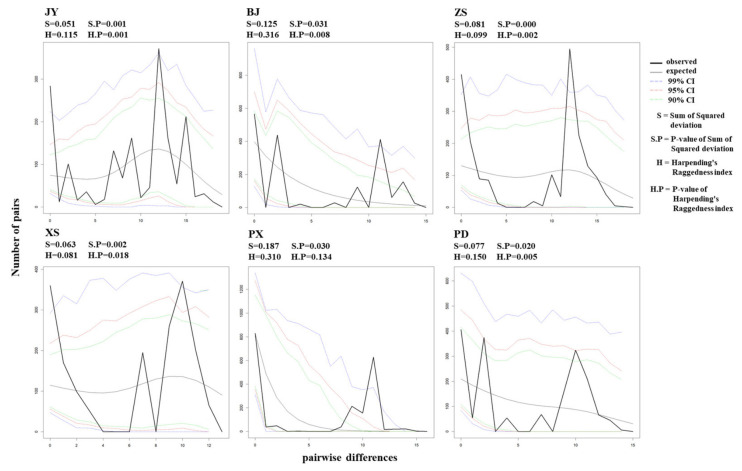
Mismatch distribution analysis based on mitochondrial DNA D-loop haplotype in six Chinese endangered indigenous chicken breeds.

Neutrality test results are presented in Table 6. *Tajima’s D* values were positive and non-significant (*p* > 0.05) for all breeds, and may suggest weak signals of population contraction or balancing processes, although none significantly deviated from neutral expectations. Xiaoshan chicken exhibited the highest *Tajima’s D* value (2.561), whereas other breeds showed values close to zero, indicating differences in mutation patterns across breeds. *Fu’s FS* values were also positive and non-significant (*p* > 0.05) across all breeds. These results did not provide strong evidence for recent demographic expansion. Mismatch distribution analysis (Figure 4) revealed a unimodal distribution for Zhongshan Shalan chicken and Pengxian Yellow chicken, suggesting possible historical population expansion. In contrast, the Jinyang Silky chicken, Bian chicken, Xiaoshan chicken, and Pudong chicken exhibited multimodal mismatch distributions (two or more peaks), with Jinyang Silky chicken displaying a particularly complex pattern, indicating more complex demographic histories.

## 4. Discussion

Local chicken breeds are assemblages of genetic resources shaped over millennia by natural selection and artificial domestication within specific ecological and socio-cultural contexts. These breeds harbor genetic variation associated with environmental adaptation, disease resistance, and distinctive production traits [19]. Genetic diversity is not only a core indicator of evolutionary potential and environmental adaptability but also a critical metric for evaluating the effectiveness of conservation programs. In this study, the six endangered chicken breeds maintained relatively high levels of maternal genetic diversity (*Hd* = 0.876, *Pi* = 0.00603). However, substantial differentiation in genetic diversity was observed among breeds, which provides important insight into the mechanisms maintaining genetic diversity in conservation populations. Historical molecular data remain limited for several endangered indigenous chicken breeds. Therefore, the present study primarily provides a current-state assessment of maternal genetic diversity under existing conservation conditions rather than a direct before-versus-after comparison of conservation outcomes.

Jinyang Silky chicken maintained relatively rich maternal genetic diversity and possessed distinctive maternal lineages compared with the other breeds. This pattern may be associated with long-term geographical isolation in the Daliangshan region and relatively limited introgression from commercial chicken populations [20]. In addition, the breed historically experienced comparatively weak artificial directional selection, which may have facilitated the retention of ancestral maternal variation. Notably, the recent successful isolation of primordial germ cells (PGCs) from Jinyang Silky chicken by the Sichuan Provincial Animal Husbandry Science Academy provides a promising technical foundation for further genetic resource preservation (https://nynct.sc.gov.cn/nynct/). In contrast, Pengxian Yellow chicken showed relatively limited maternal genetic diversity, which may be associated with historical population bottlenecks and long-term small population size [21]. Such extremely small population sizes likely produced strong bottleneck and founder effects, leading to reduced genetic variation. Similarly, Bian chicken and Pudong chicken also showed relatively low diversity, aligning with genomic assessments based on SNP chips [22]. A study evaluating conservation effectiveness in Taihang chicken similarly found significant differences in genetic structure among conservation populations, with some populations even including full-sibling individuals, highlighting the critical impact of scientifically sound conservation schemes on maintaining genetic diversity [23]. This finding serves as a warning: ex situ conservation models may still experience hidden losses of genetic diversity under small population conditions.

Haplotype composition and distribution patterns provide a crucial window into population genetic dynamics. The haplotype network analysis supported the complex maternal origin of Chinese indigenous chickens and was generally consistent with previous mtDNA studies reporting multiple maternal lineages in domestic chickens [10,24]. The star-like network topology centered on several common haplotypes may reflect historical population expansion and widespread ancestral maternal lineages. However, because neutrality tests were not statistically significant, these demographic inferences should be interpreted cautiously. Population differentiation analyses indicated that maternal genetic variation was predominantly distributed within breeds rather than among breeds, suggesting incomplete maternal lineage separation among these indigenous chicken populations. The relatively weak differentiation observed among several breed pairs may reflect shared ancestral maternal origins and historical germplasm exchange during domestication and conservation processes. Research on Ethiopian indigenous chickens warns that introgression from exotic bloodlines may lead to genetic dilution and loss of uniqueness in local genetic characteristics [25]. Therefore, uncontrolled germplasm exchange among conservation farms may gradually lead to genetic homogenization, diminishing the overall diversity of indigenous chicken genetic resources.

A cautionary case from the Taihang chicken conservation evaluation study deserves attention [23]. In that study, due to non-standard practices during the avian leukosis purification process, some families were eliminated, leading to the introduction of a certain number of full-sibling individuals. This consequently altered the genetic structure of the two conserved populations and increased their genetic distance. This finding suggests that even different conservation populations of the same breed may develop significant genetic structural differentiation due to variations in management practices. Therefore, it is recommended to establish monitoring mechanisms for genetic exchange among conservation populations. In particular, molecular markers should be used to track germplasm introductions into core conservation populations to avoid the erosion of genetic uniqueness caused by conservation hybridization.

Based on the above discussion, several recommendations can be proposed for the conservation management of endangered local chicken breeds. For Jinyang Silky chicken, possessing the highest proportion of private haplotypes (60%) and unique haplogroups (F and G), priority should be given to maintaining its genetic integrity by establishing independent core conservation populations and avoiding admixture with other breeds. For Zhongshan Shalan chicken [26] and Xiaoshan chicken [27], with relatively high genetic diversity levels and established industrial development foundations, genetic monitoring should be strengthened while promoting conservation through utilization. In contrast, Bian chicken [28], Pudong chicken [29], and Pengxian Yellow chicken [30], which exhibit relatively low genetic diversity, require close monitoring of inbreeding levels and careful management of breeding populations.

The present study employed mitochondrial DNA D-loop sequences because this marker system is widely used in studies of indigenous chicken genetic resources and facilitates comparison with previously published maternal lineage data. In addition, mtDNA analysis provides a relatively cost-effective approach for the large-scale preliminary assessment of maternal genetic diversity in endangered populations. However, mitochondrial DNA represents only a single maternally inherited genetic locus and therefore cannot fully capture genome-wide diversity patterns. In contrast, nuclear molecular markers such as microsatellites, SNP chips, and whole-genome resequencing reflect biparental inheritance and are more informative for evaluating admixture, inbreeding, effective population size, selection signatures, and overall genomic conservation status. Consequently, mtDNA-based diversity patterns may not fully correspond to genome-wide genetic variation. Future studies integrating multiple genomic approaches will therefore be essential for comprehensive conservation genetic evaluation of endangered indigenous chicken breeds.

## 5. Conclusions

Based on mitochondrial DNA D-loop sequences, this study systematically evaluated the maternal genetic diversity and population structure of six endangered indigenous chicken breeds in China. Under current conservation conditions, these breeds collectively retain a moderate level of maternal genetic diversity based on mitochondrial markers. Among them, Jinyang Silky chicken exhibited comparatively high haplotype and nucleotide diversity and a relatively complex maternal genetic structure, whereas Bian chicken and Pengxian Yellow chicken showed lower levels of diversity and more centralized haplotype distributions. Haplotype network and population differentiation analyses indicated that distinct maternal lineages have not formed among the six breeds and several high-frequency haplotypes are widely distributed across breeds. AMOVA results demonstrated that most genetic variation occurs within breeds, with moderate differentiation among breeds. Neutrality tests did not reveal significant departures from neutral expectations, although mismatch distribution analysis suggested possible population expansion in Zhongshan Shalan chicken and Pengxian Yellow chicken. Nevertheless, because mitochondrial DNA reflects only maternal inheritance, these findings cannot fully represent nuclear genomic diversity or overall conservation status. Future studies integrating genome-wide molecular markers will be necessary for more comprehensive conservation assessments.

## Figures and Tables

**Table 1:** Information of six endangered indigenous chicken breeds in China.

Breed	Endangered Level	Origin	Number
Bian chicken (BJ)	endangered	Youyu, Shanxi	61
Jinyang Silky chicken (JY)	endangered	Liangshan, Sichuan	60
Pudong chicken (PD)	endangered	Pudong, Shanghai	60
Xiaoshan chicken (XS)	endangered	Xiaoshan, Zhejiang	60
Zhongshan Shalan chicken (ZS)	endangered	Zhongshan, Guangdong	63
Pengxian Yellow chicken (PX)	critically endangered	Pengzhou, Sichuan	64

**Table 2:** Genetic diversity parameters of six indigenous chicken breeds in China.

Breed	Number of Polymorphic Sites (*S*)	Haplotype Diversity (*Hd*)	Nucleotide Diversity (*Pi*)	Average Number of Nucleotide Differences (*K*)
BJ	18	0.691 ± 0.046	0.0041 ± 0.00044	5.095
JY	31	0.840 ± 0.029	0.0069 ± 0.00028	8.530
PD	21	0.771 ± 0.040	0.0044 ± 0.00044	5.371
PX	23	0.589 ± 0.056	0.0047 ± 0.00042	5.750
XS	15	0.797 ± 0.019	0.0049 ± 0.00023	5.982
ZS	27	0.788 ± 0.038	0.0060 ± 0.00031	7.477

**Table 3:** Unique and shared haplotypes and haplogroup distribution of six indigenous chicken breeds in China.

Breed	Number of Haplotypes (*H*)	Unique Haplotype ^(1)^	Shared Haplotype ^(1)^	Haplogroup					
				A	B	C	E	F	G
BJ	6	3.3(1)	96.7(5)	26	3		70		
JY	12	60.0(7)	40.0(5)	28	27		7	3	35
PD	7	15.0(1)	85.0(6)	67	3	5	25		
PX	8	9.4(4)	90.6(4)	61	3	6	30		
XS	6	40.0(3)	60.0(3)	53		22	25		
ZS	14	11.1(5)	88.9(9)	2	40	8	51		

^(1)^ Percentages indicate the proportion of individuals carrying unique or shared haplotypes, whereas the values in parentheses indicate the number of haplotypes.

**Table 4:** Analysis of molecular variance (AMOVA) for six endangered indigenous chicken breeds in China.

Source of Variation	df	Sum of Squares	Variance Components	Percentage of Variation
Among populations	5	211.92	0.639 Va	16.59
Within populations	362	1162.85	3.212 Vb	83.41
Total	367	1374.77	3.851	
Fixation Index	*Fst* = 0.166			

**Table 5:** Population pairwise *Fst* value (above the diagonal) and gene flow (below the diagonal) for comparisons between six Chinese endangered indigenous chicken breeds.

Breed	BJ	JY	PD	PX	XS	ZS
BJ		0.245	0.238	0.184	0.172	0.130
JY	1.540		0.200	0.199	0.204	0.115
PD	1.602	2.004		0.005 ^+ (1)^	0.041	0.222
PX	2.214	2.019	92.659		0.035	0.197
XS	2.402	1.957	11.750	13.644		0.186
ZS	3.350	3.848	1.750	2.035	2.188	

^(1)^ The plus sign indicates that the *Fst* significance test was not significant (*p* > 0.05).

**Table 6:** Analysis of neutrality test for six endangered indigenous chicken breeds in China.

Neutrality Test	BJ	JY	PD	PX	XS	ZS
*Tajima’s D*	0.991	0.921	0.601	0.569	2.561	0.972
*Tajima’s D p-value*	0.873	0.858	0.753	0.749	0.995	0.878
*Fu’s FS*	7.419	4.613	6.247	5.470	8.609	2.218
*Fu’s FS p-value*	0.981	0.905	0.967	0.943	0.982	0.806

## Data Availability

All sequences described in the study have been deposited in GenBank. Accession numbers provided in the study range from PZ189266 to PZ189633.

## Abbreviations

The following abbreviations are used in this manuscript:

mtDNA	mitochondrial DNA
AMOVA	analysis of molecular variance
Hd	haplotype diversity
Pi	nucleotide diversity
K	average number of nucleotide differences
S	number of polymorphic sites
H	number of haplotypes
